# Serial change of neutrophil extracellular traps in tracheal aspirate of patients with acute respiratory distress syndrome: report of three cases

**DOI:** 10.1186/s40560-020-00444-5

**Published:** 2020-04-10

**Authors:** Masahiro Ojima, Norihisa Yamamoto, Tomoya Hirose, Shigeto Hamaguchi, Osamu Tasaki, Takashi Kojima, Kazunori Tomono, Hiroshi Ogura, Takeshi Shimazu

**Affiliations:** 1 Department of Traumatology and Acute Critical Medicine, Osaka University Graduate School of Medicine, Suita, Osaka, Japan; 2 Division of Infection Control and Prevention, Osaka University Hospital, Suita, Osaka, Japan; 3 Acute and Critical Care Center, Nagasaki University Hospital, Nagasaki, Japan; 4 Central Laboratory for Clinical Investigation, Osaka University Hospital, Suita, Osaka, Japan

**Keywords:** Neutrophil extracellular traps (NETs), Acute respiratory distress syndrome (ARDS), Histone, Citrullinated histone

## Abstract

**Background:**

Neutrophil extracellular traps (NETs) are fibrous structures released from activated neutrophils. NET formation has been reported to be associated with acute respiratory distress syndrome (ARDS). However, there are no reports dealing with serial changes of NET formation in tracheal aspirate of ARDS patients.

**Case presentation:**

We report three cases of ARDS. Case 1 is a 69-year-old man with necrotizing fasciitis of the buttocks, case 2 is a 49-year-old woman with extensive burns (80% of total body surface), and case 3 is a 73-year-old woman with severe bacterial pneumonia. We found abundant expression of citrullinated histone H3 (Cit-H3) and the formation of NETs at the onset of ARDS in all cases. The amounts of Cit-H3 and NETs decreased with the amelioration of respiratory failure in cases 1 and 2. In case 2, the amounts of Cit-H3 and NETs increased with aggravation of infection and respiratory status. In case 3, the abundant expression of Cit-H3 and NETs persisted; the patient did not recover from ARDS and eventually died. Cit-H3 and NETs were found in tracheal aspirates even if the patients had no direct injury to the lung as in cases 1 and 2.

**Conclusions:**

In these three cases, the formation of NETs was observed in tracheal aspirate of patients with ARDS by either direct or indirect insults to the lung. The amount of NET formation changed dynamically over the clinical course of each patient.

## Background

Neutrophil extracellular traps (NETs) are fibrous structures released from activated neutrophils. The main components of NETs are deoxyribonucleic acid (DNA), histones, and intracellular antibacterial proteins such as neutrophil elastase and myeloperoxidase [1]. Citrullination of histone H3 leads to the formation of NETs by decondensing chromatin, which is a marker of active NET formation [2]. NETs rapidly trap and kill pathogens by antibacterial proteins and histone toxicity [1]. In addition, NETs lead to the recruitment of neutrophils and induce clotting within blood vessels [3]. These mechanisms prevent bacteria from disseminating hematogenously to other tissues, but they can also cause endothelial and tissue damage through inflammation and ischemia [4]. The formation of NETs is stimulated by contact with pathogens such as bacteria or fungi and by various factors such as inflammatory stimuli (damage-associated molecular patterns, cytokines, or activated platelets) or chemical compounds [5]. The clearance of NETs is processed by DNase and macrophages [6].

Acute respiratory distress syndrome (ARDS) remains one of the major challenges in the acute onset of respiratory failure. Injury is initiated by either direct or indirect insults to the alveolar structure of the lung. It is characterized pathologically by diffuse alveolar damage, fibrin deposition, and excessive infiltration of neutrophils and macrophages into the alveolar space. Large amounts of toxic mediators (neutrophil elastase, myeloperoxidase, histones, and reactive oxygen species) are also detected in the alveolar spaces [7]. Because the components of intraalveolar fluid from ARDS patients are similar to the component elements of NETs, the association between ARDS and NETs has been focused on.

Previous reports suggested that NET formation was associated with ARDS and was also related to the severity of ARDS [8–12]. These reports assessed the amount of NETs by quantifying DNA and histones in alveolar lavage fluid, lung tissue, or plasma by cross-sectional analysis compared to controls or healthy people. The serial change of NET formation in ARDS patients is unclear. In addition, sampling by alveolar lavage or lung biopsy is an invasive medical procedure that is difficult to perform in patients with severe respiratory failure. Thus, it is necessary to perform a less invasive method to identify NETs in ARDS patients. Tracheal aspirate may be a surrogate sample for assessing NET formation in ARDS patients. However, there have been no reports, to our knowledge, of NET formation being observed in tracheal aspirates of ARDS patients. Here, we report three cases of ARDS patients with morphological assessment of NET formation in tracheal aspirates and their clinical courses.

## Case presentation

We identified NETs by following the method used in a previous study [13]. Extracellular components including DNA and histone H3, which are the major components of NETs, in tracheal aspirate were simultaneously detected using immunostaining in patients with ARDS. We stained DNA with 4′,6-diamidino-2-phenylindole (DAPI; Invitrogen, USA) and histone H3 with anti-human histone H3 mouse monoclonal antibody (MAB Institute, Inc., Japan). We also identified active NET release by detecting citrullinated histone H3 (Cit-H3) using anti-human Cit-H3 rabbit polyclonal antibody (Abcam, UK). We diagnosed ARDS according to the 2012 Berlin definition [14]. We investigated 20 cases diagnosed as ARDS from October 2013 to January 2015 and present here three cases representative of these 20 cases.

### Case 1

**Fig. 1:**
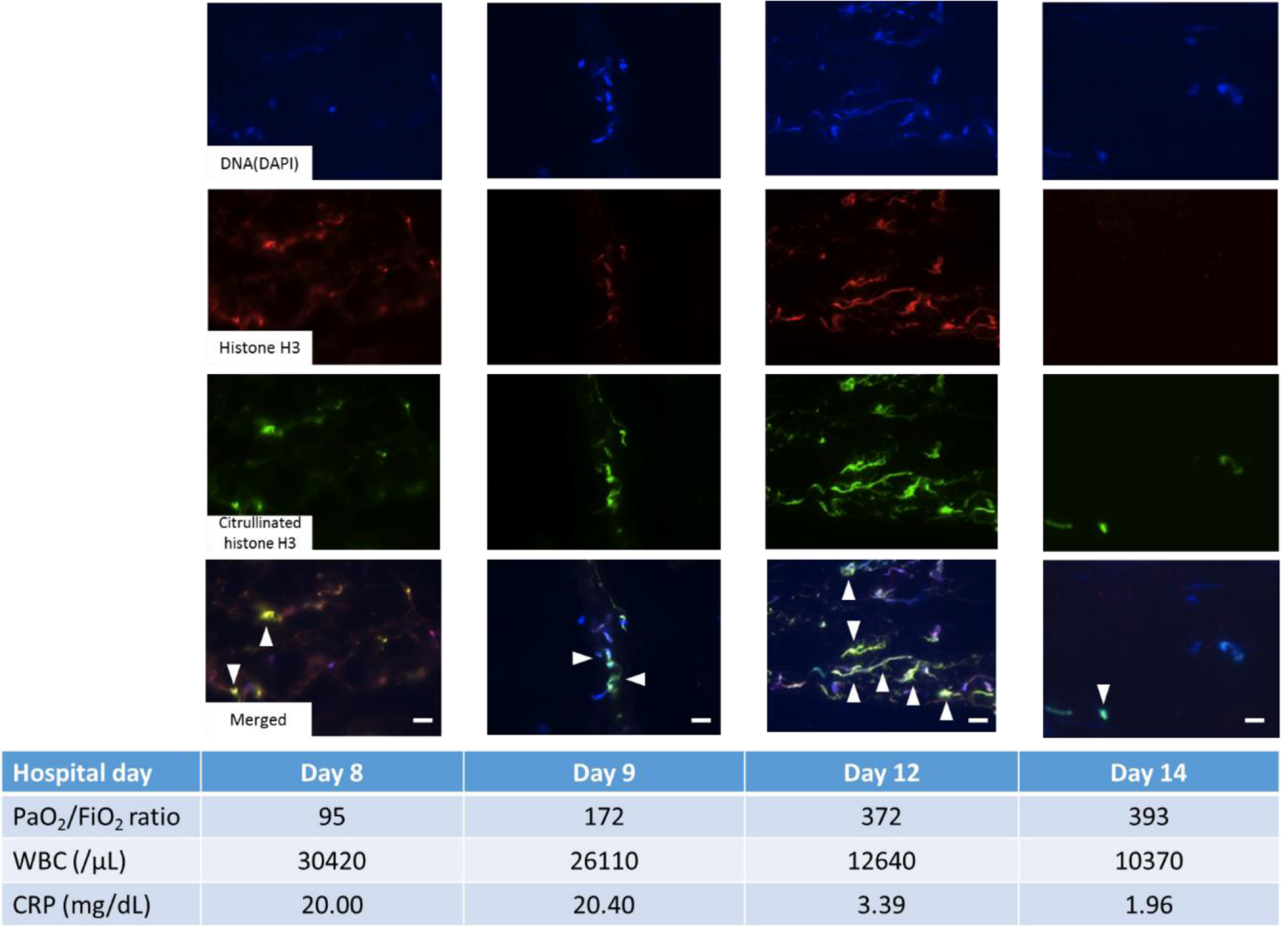
Time course images of immunostaining of tracheal aspirates and changes of PaO_2_/FiO_2_ ratio, white blood cell count (WBC), and C-reactive protein (CRP) in case 1. Methylprednisolone (150 mg/day) was given intravenously from hospital days 8 to 18. Triple staining was performed with 4′,6-diamidino-2-phenylindole (DAPI), anti-human histone H3 mouse monoclonal antibody, and anti-human Cit-H3 rabbit polyclonal antibody. Blue: DAPI; red: histone H3; green: citrullinated histone H3. White arrowheads indicate NETs. Scale bar = 30 μm

A 69-year-old man with a history of diabetes mellitus was admitted with necrotizing fasciitis of the buttocks. He was suffering from severe septic shock and respiratory distress for which he was promptly intubated and underwent incisional drainage and debridement along with the administration of broad-spectrum antibiotics (penicillin G, meropenem, and clindamycin). However, the control of his infection did not progress, and his respiratory condition worsened. On hospital day 8, his PaO_2_/FiO_2_ ratio dropped below 100, and he was diagnosed as having severe ARDS. Gram staining of tracheal aspirate on hospital day 8 showed no bacteria, but a culture of the same sample confirmed a small amount of *Corynebacterium* species. His respiratory status improved gradually with amelioration of the infection by daily debridement and irrigation of his wounds. He recovered from ARDS by hospital day 14 and was weaned from ventilator support on hospital day 19. Immunostaining of his respiratory samples showed abundant expression of Cit-H3 and NETs on hospital days 8, 9, and 12. However, Cit-H3 was rarely observed and NETs almost completely disappeared by hospital day 14 (Fig. 1).

### Case 2

**Fig. 2:**
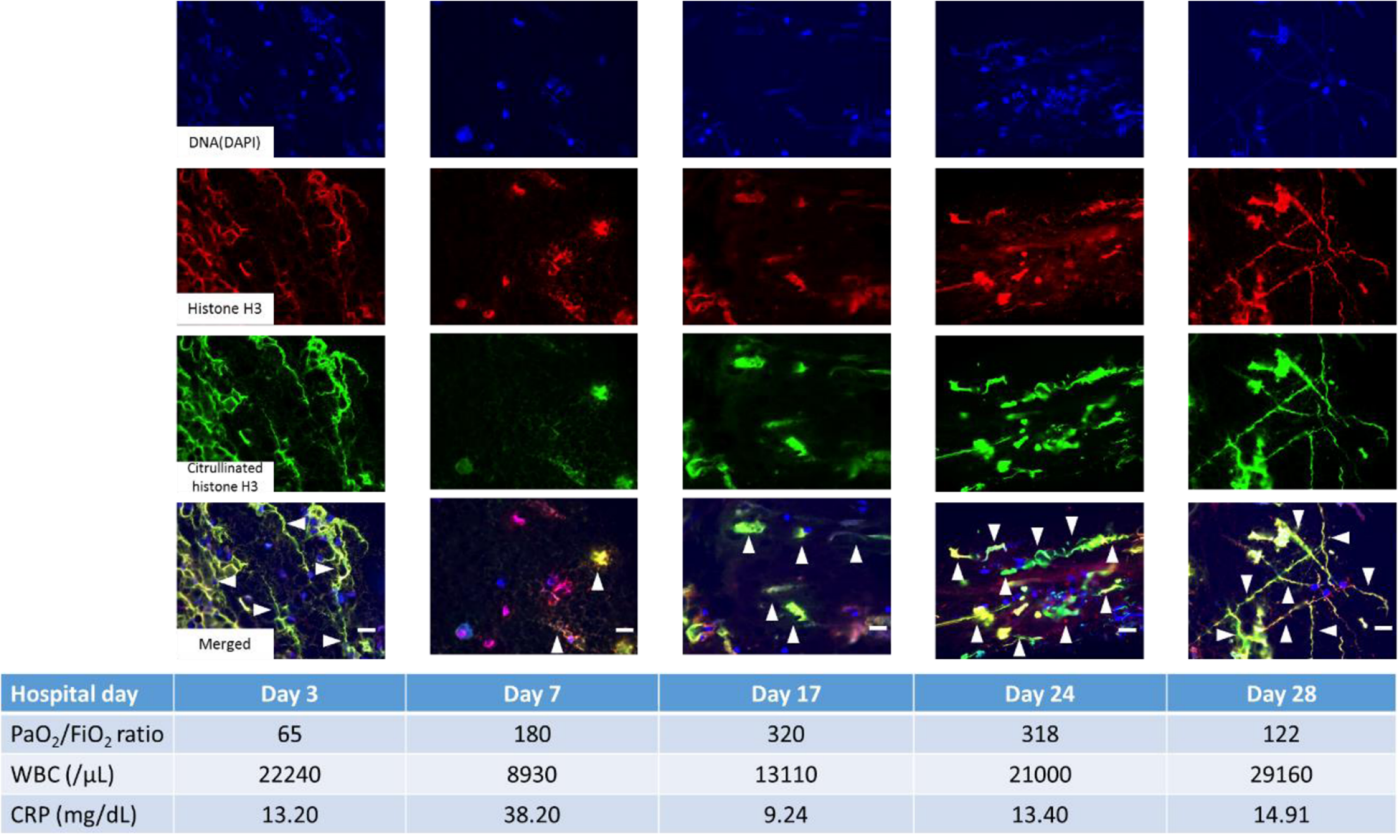
Time course images of immunostaining of tracheal aspirates and changes of PaO_2_/FiO_2_ ratio, white blood cell count (WBC), and C-reactive protein (CRP) in case 2. Steroids were not given over the course of hospitalization. Blue: DAPI; red: histone H3; green: citrullinated histone H3. White arrowheads indicate NETs. Scale bar = 30 μm

A 49-year-old woman was admitted with extensive burns (80% of total body surface). She had full-thickness burns on nearly her entire trunk, but she had not suffered inhalation burn. She was promptly intubated and treated with sufficient doses of crystalloid fluid. She was diagnosed as having compartment syndrome and underwent escharotomy of the chest, abdomen, and limbs. Her respiratory status rapidly worsened and progressed to severe ARDS on hospital day 3. We could not find any organisms by Gram staining or culture of her tracheal aspirate on hospital day 3. She underwent initial escharectomy on hospital day 3 and also escharectomy and split-thickness skin grafting several times up to hospital day 27. Her respiratory status gradually ameliorated, and she recovered from ARDS by hospital day 17. She developed a high fever on hospital day 24, and the burn wound was found to be infected with *Pseudomonas aeruginosa* and methicillin-resistant *Staphylococcus aureus*. Her respiratory condition worsened again, leading to moderate ARDS on hospital day 25. Gram staining and culture of the tracheal aspirate on hospital days 24 and 28 did not show any pathogens. She was treated with antibiotics (ceftazidime and vancomycin) and recovered from infection and respiratory failure by hospital day 30. Immunostaining of her tracheal aspirate showed abundant expression of Cit-H3 and NETs on hospital day 3. The expression of Cit-H3 and NETs were found to diminish on hospital days 7 and 17, but they increased significantly on hospital days 24 and 28 (Fig. 2).

### Case 3

**Fig. 3:**
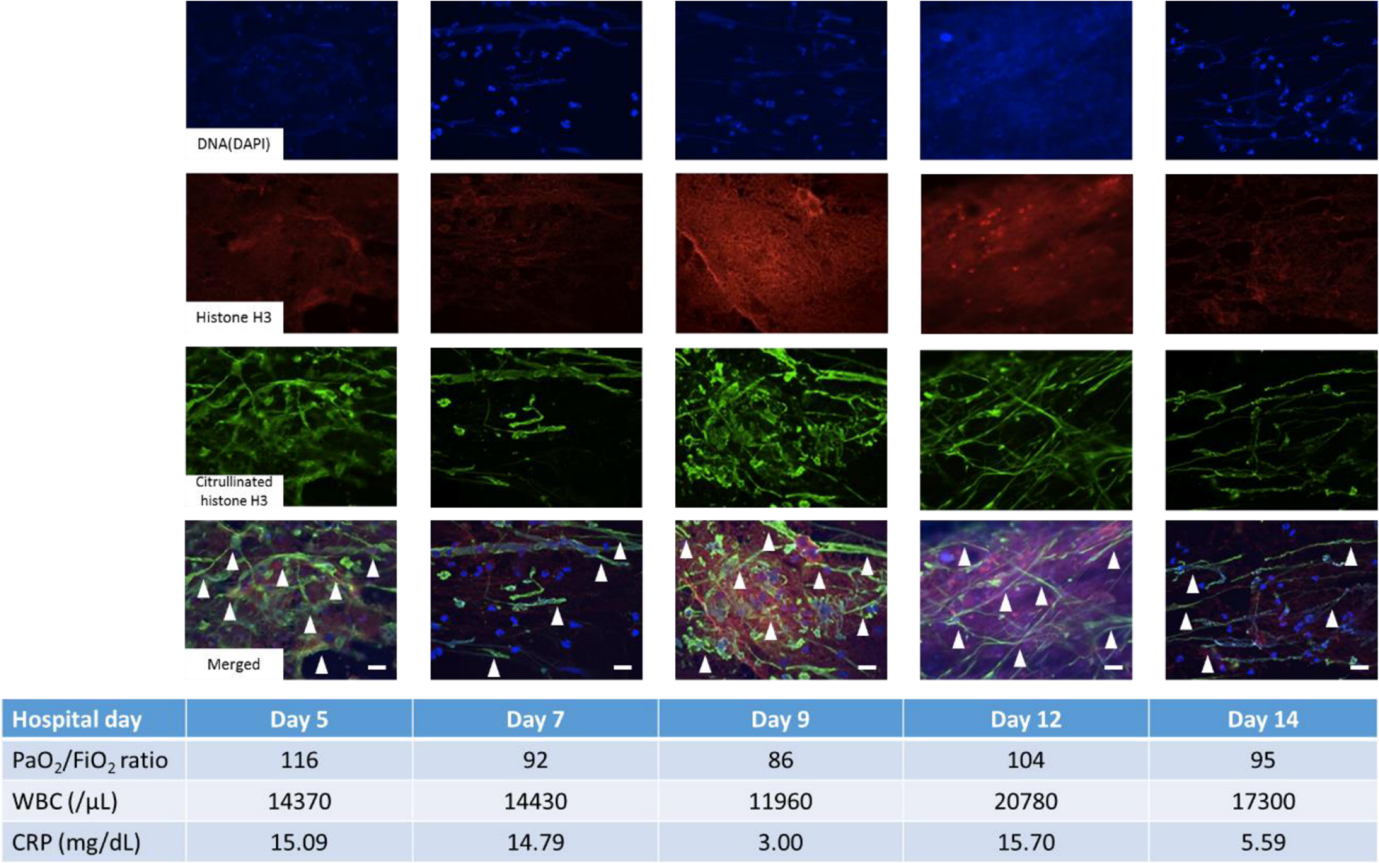
Time course images of immunostaining of tracheal aspirates and changes of PaO_2_/FiO_2_ ratio, white blood cell count (WBC), and C-reactive protein (CRP) in case 3. Methylprednisolone (1000 mg/day) was given intravenously from hospital days 6 to 8, and prednisolone (100 mg/day) was given through feeding tube from hospital days 4 to 8. Blue: DAPI; red: histone H3; green: citrullinated histone H3. White arrowheads indicate NETs. Scale bar = 30 μm

A 73-year-old woman with a history of malignant lymphoma was admitted with bacterial pneumonia. We administered high-flow oxygen and broad-spectrum antibiotics (meropenem and vancomycin). However, her respiratory status worsened and progressed to severe ARDS on hospital day 5. Gram staining of her tracheal aspirate on the day of admission showed Gram-negative rods and Gram-positive cocci with phagocytosis by leucocytes, and culture of the same sample confirmed the presence of *Pseudomonas aeruginosa* and *Corynebacterium* species. Although she underwent intensive care, her respiratory status never improved, and she died on hospital day 16. Immunostaining of her sputum showed abundant expression of Cit-H3 and NETs from hospital day 5 that persisted until hospital day 14 (Fig. 3).

## Discussion

In these three patients, we found abundant expression of Cit-H3 and NETs in tracheal aspirates at the onset of ARDS. The amounts of Cit-H3 and NETs decreased with the amelioration of respiratory failure in cases 1 and 2, whereas in case 2, they increased with aggravation of the burn wound infection that was accompanied by worsening of her respiratory status. In case 3, the expression of Ci-H3 and NETs was persistent, with the patient failing to recover from ARDS and eventually dying from respiratory failure. In addition, NETs were observed in the tracheal aspirates of all three patients even if they suffered no direct injury to the lung as in cases 1 and 2. These findings indicate that (1) the formation of NETs may be observed in the tracheal aspirate of patients with ARDS, (2) the amount of NETs formation may dynamically change over the clinical course of ARDS, and (3) NETs in tracheal aspirate may be induced not only by direct injury to lung tissue but also by severe insult to other tissues.

NETs have been detected in various sites (bronchoalveolar lavage fluid, lung tissue, and plasma) in ARDS patients [8–12]. The timing of sampling was heterogenous in these previous reports, and thus, the timing of the onset and transition of NET formation in these ARDS patients were unclear. In the present report, we found NET formation in tracheal aspirates on the day ARDS was diagnosed. The expression of NETs continued during the ARDS state and decreased along with the recovery from ARDS. We previously reported that NETs were observed during acute respiratory infection in tracheal aspirates of intubated patients [13, 15]. NETs in tracheal aspirates might be widely found in acute inflammatory lung diseases.

The amount of NETs appeared to increase after clinical improvement in cases 1 (on day 12) and 2 (on day 17). We previously reported that the length of NETs in tracheal aspirates initially increased and then shortened during acute respiratory infection, and this correlated with the level of pro-inflammatory cytokines (such as interleukin-8 or CXC ligand-2) in the blood [13]. The association between interleukin-8 and NETs was also reported in ventilator-associated pneumonia [9]. The amount of NET expression in bronchial aspirates might be associated with the increase of pro-inflammatory cytokines even in ARDS patients. It was also reported that the formation of suicidal NETs induced by various stimuli occurred within 3–4 h in vitro [16]. However, the timing of NET formation triggered by stimuli in vivo is unclear. As we reported previously, the maximum amount of NET release was observed on the day following the development of bacterial pneumonia, whereas the level of pro-inflammatory cytokines in the blood was most abundant on the day of onset [13]. The release of NETs via pro-inflammatory stimuli might be delayed in critical situations in humans when compared with that in vitro. Further study is needed to confirm these suppositions.

The association between NETs and ARDS in humans has been reported in bacterial pneumonia [8], ventilator-associated pneumonia [9], transfusion-related acute lung injury [10], sepsis [11], and trauma [12]. These reports all suggested an association between the quantity of NETs and the severity of ARDS: in other words, “the more NETs, the more severe ARDS.” In our patients, the progression or regression of NETs in tracheal aspirates paralleled the patients’ respiratory status, and the persistent release of NETs resulted in a poor outcome in case 3. Observed changes of NET release in aspirate can be a marker of ARDS severity and predictor of the clinical course. They also imply that controlling NET formation might contribute to the control of ARDS. Previous reports suggested that not only excess expression of NET release but also reduced clearance of NETs by macrophages and DNase were observed in ARDS patients [11, 17]. Controlling NET formation by inhibiting inflammatory stimuli or enhancing the clearance function might be new therapy for ARDS. Further accumulation of basic and clinical observations is warranted to elucidate this point.

We previously reported the presence of NETs in the tracheal aspirates of patients with acute respiratory infections [13, 15]. We also identified NETs in the bloodstream in intubated patients, and NETs were more frequently detected in “the presence of bacteria in tracheal aspirates” group [18]. These findings suggested that the presence of bacteria in the respiratory tract may be associated with NET formation in the tracheal aspirates and blood. Other reports suggested that infection was a major factor inducing NET formation but that infection was not necessary to trigger their release. NET release is induced by various stimuli: microorganisms, damage-associated molecular patterns, pathogen-associated molecular patterns, cytokines, and chemokines [19]. NET release occurred in our patients with not only direct lung injury caused by microbial pneumonia but also indirect injury due to severe insults of other tissues. This finding suggests that NETs in tracheal aspirates could be formed by stimuli other than direct bacterial effects and may help in understanding the pathology of ARDS in critical illness.

Finally, there may be selection bias due to the nature of case reports. Additional accumulation of cases is needed to further evaluate our findings in the future.

## Conclusion

The formation of NETs was observed in the tracheal aspirate of three patients with ARDS by either direct or indirect insults to the lung. The amount of NET formation changed dynamically over the clinical course of each patient. However, the cases presented here are inadequate to clarify the association between NETs and ARDS, and further analysis of cases will be needed to elucidate our findings.

## Data Availability

All data generated or analyzed during this study are included in this published article.

## Abbreviations

ARDS: Acute respiratory distress syndrome
Cit-H3: Citrullinated histone H3
DNA: Deoxyribonucleic acid
NETs: Neutrophil extracellular traps
